# Fungal backpackers—the mycobiome of *Ips typographus* after more than 80 years of research

**DOI:** 10.3389/fmicb.2025.1695278

**Published:** 2026-01-21

**Authors:** Flavius Popa, Joern Buse, Peter H. W. Biedermann, Vienna Kowallik

**Affiliations:** 1 Black Forest National Park, Seebach, Germany; 2 Chair for Forest Entomology and Protection, University of Freiburg, Freiburg, Germany

**Keywords:** bark beetle, fungi, mycobiome, plant pathogens, plant–fungus–insect interactions, vector, symbionts

## Abstract

The European spruce bark beetle, *Ips typographus*, primarily colonizes Norway spruce and plays a pivotal ecological and economic role across Eurasia. Over decades, numerous studies have identified fungal species associated with *I. typographus* to comprehend their composition and relationships with the beetle and its tree host. The literature reveals a complex, diverse, and dynamic fungal community posing challenges in identifying consistent patterns. To enhance our understanding of the relationships in this tri-trophic system, a comprehensive overview of fungal associates is required. In this meta-analysis, we summarize the fungal species that have been found in association with *I. typographus*. Across 58 studies conducted over the last 80 years, 712 fungal species have been documented. Among these, 14 phytopathogenic species have been consistently recorded throughout the beetle’s distribution range in Europe and Asia, therefore considered being part of its core mycobiome. A further 150 species were documented in only one part of the beetles’ distribution range, and were classified as potential members of the core mycobiome. A significant proportion of the fungal assemblages were identified as passively associated species, constituting 77% of the total. We emphasize and engage in a critical discussion of the biases reflected in this data set, specifically those arising from the scientific methods employed and the sampled geographic areas. The majority of studies incorporated within this meta-analysis (*n* = 52) are based on conventional fungal culturing techniques with few recent publications (*n* = 6) incorporating modern molecular methods. At this point, the data suggest that the integration and complementary use of both methods may provide a more comprehensive representation of the mycobiome. Most studies have focused on Central and Northern Europe, with only six in Asia, leading to a significant data imbalance across the beetle’s range. Hence, the data here provide a snapshot of current research, with expectations for further development with future studies. This identified diverse array of fungi within the beetle’s mycobiome underscores the dynamic interactions between host trees, bark beetles, and their associated fungal community, highlighting their crucial roles in the beetle’s ecological success and illustrating its deep integration into a complex fungal ecosystem.

## Introduction

Plant-insect interactions represent a diverse, yet fundamental element in ecological networks, crucial for maintaining biodiversity and ecosystem functions (Raffa et al., 2008; Whitehill et al., 2023). Throughout evolutionary history, the often-overlooked kingdom of fungi has developed intricate relationships with both plant and insect hosts, playing a pivotal role in shaping these inter-kingdom interactions (Frago et al., 2012; Birkemoe et al., 2018). In the case of wood-boring insects such as bark beetles, the three kingdoms—plants, insects, and fungi—are intricately linked (e.g., Hofstetter et al., 2015a; Hofstetter et al., 2015b; Kandasamy et al., 2023). Here, especially the mass outbreaks of bark beetles and their phytopathogenic fungal symbionts, that are under certain circumstances able to infest and kill healthy trees, are a topic of global interest and concern (Biedermann et al., 2019; Netherer et al., 2021; Whitehill et al., 2023). While bark beetle - tree interactions, such as those of conifers with *Dendroctonus* spp. in the US or *Ips* spp., have been extensively studied due to their significant impact on forest ecosystems (Raffa et al., 2015; Kirkendall et al., 2015; Netherer et al., 2021; Fettig et al., 2022), the multifaceted roles and whole diversity of fungal symbionts—ranging from plant parasites to beneficial mutualists and antagonists—remain usually underexplored (but see Kirschner et al., 2001).

Historically, the partnership between bark beetles and their fungal associates was first recognized almost 200 years ago (e.g., Hartig, 1844; Neger, 1908; Leach et al., 1935) and broadly studied since the 1930s (Grosmann, 1931; Mathiesen, 1950). Francke-Grosmann was the first to discover morphological adaptations of fungal spores (e.g., sticky surfaces), which specialize them for symbiotic functions and beetle dispersal (Grosmann, 1931). Corresponding morphological adaptations, like specific structures for spore dispersal (i.e., mycetangia) were described for some beetles in the following (Nunberg, 1951; Francke-Grosmann, 1956; Batra, 1963). While the degree and mode of the associations vary tremendously across bark beetle species (e.g., Lieutier et al., 2009; Barta et al., 2020; Hulcr et al., 2020; Mayers et al., 2022), research has evidenced considerable specialization of fungal taxa corresponding to distinct bark beetles, and demonstrated a co-evolutionary history between certain bark beetle clades and specific fungal taxa (Chapela et al., 1994; Harrington, 2005; Peris et al., 2021; Francke-Grosmann, 1967). Those can be recognized as having evolved morphological adaptations in both the fungi (e.g., enlarged, nutritional spores) and the beetles (e.g., mycetangia) (Biedermann and Vega, 2020). So far, we know of at least 14 independent evolutionary origins of obligate mutualisms in bark beetles (Six and Biedermann, 2023).

Notably, while some fungi act negatively as pathogens or resource competitors to the beetles (Wegensteiner et al., 2014; Wegensteiner and Weiser, 2004), mutualistic fungi confer substantial benefits to their beetle hosts by altering their microenvironment in various ways (Hofstetter et al., 2015a; Hofstetter et al., 2015b; Six, 2013). They can be implicated in exhausting and detoxifying tree defenses (Lieutier et al., 2009; Zhao et al., 2019), outcompeting pathogenic or competing microbes (Strid et al., 2014) and benefiting the nutritional requirements of developing beetles by remobilizing nutrients back to the phloem, concentrating nitrogen and phosphorous and supplying essential dietary nutrients (Ayres et al., 2000; Bentz and Six, 2006; Davis et al., 2019; Six, 2020; Six and Elser, 2020; Six and Wingfield, 2011). Furthermore, some volatile compounds released by fungi are not only attractive to beetles but can also play a critical role in facilitating intraspecific communication among bark beetles when colonizing new tree hosts (i.e., aggregation pheromones), underscoring the ecological complexity of these relationships (Kandasamy et al., 2019; Tanin et al., 2021; Zhao et al., 2019). Host-plant-fungal-insect interactions are inherently influenced by environmental factors such as temperature and humidity. Climate change, particularly drought-induced stress, has impacted those interactions, for example between bark beetles and conifers, by reducing host tree vitality and resilience and enabling more beetle generations per reproduction season due to longer and warmer summer periods (Hofstetter et al., 2007; Athanassiou et al., 2017; Biedermann et al., 2019; Netherer et al., 2021). Amongst these interaction complexes, *Ips typographus*, the Eurasian spruce bark beetle, which predominantly colonizes Norway spruce (*Picea abies*), stands out due to its wide distribution and economic significance across Europe and parts of Asia, including trans-Palearctic regions such as Siberia, China, Korea, and Japan (Gregoire and Evans, 2004; Wermelinger, 2004; Hlásny et al., 2021).

The fungal community associated with *I. typographus* is highly diverse and includes plant pathogenic fungi, saprobionts, insect pathogens, and mycoparasites, all with the potential to influence outbreak dynamics (Zhao et al., 2019; Persson et al., 2009). The interaction between *I. typographus* and fungi is characterized by a notable assemblage of ascomycete species, particularly from the genera *Ophiostoma* and *Grosmannia* (both Ophiostomatales), as well as *Ceratocystis* and *Endoconiophora* (Microascales). These fungi are common facultative associates of the beetle, enhancing its fitness in various ways. However, among this fungal list, *Endoconidiophora polonica* (Microascales) stands out as a highly specialized species, not known to exist independently of bark beetles (Kirschner, 1998; Kirisits, 2004). While *I. typographus* exhibits no obligate symbiotic relationship with any specific fungus, it likely benefits from a flexible fungal dependency that can be satisfied by multiple species (Schebeck et al., 2023; Six and Biedermann, 2023). This is in strong contrast to other aggressive bark beetles for example in the genus *Dendroctonus*, which maintain obligate and species-specific fungal relationships, resulting in a less diverse symbiont community (Bracewell and Six, 2015). The factors driving the diversity of *I. typographus’s* symbiont community remain unclear, though it is probable that factors such as their host spectrum (attacking both healthy and vitality reduced trees), high colonization densities, polygynous mating system, broad geographical distribution and other co-colonizing bark beetle species may play a role (Kirisits, 2004; Biedermann et al., 2019; Six and Biedermann, 2023).

In this context, while progress has been made in cataloging *I. typographus*-associated fungi, critical questions persist. For instance, the extent to which the beetle’s geographic distribution aligns with that of its fungal partners, and the distinction between passively and actively dispersed fungal species, remain underexplored. This study provides a comprehensive overview of all fungal species that have been found in association with *I*. *typographus* across its range of distribution from Western to Northern Europe and the Asian Far East. Our aim is to identify core taxa that are consistently present throughout the beetle’s entire distribution range and to distinguish the potentially associated mycobiome, which may have been underrepresented due to research biases in existing literature, from passively associated species that serve as temporary environmental transients, enhancing the ecological understanding of the roles fungi play in this symbiotic relationship.

## Materials and methods

### Literature search and meta-analysis

An extensive literature research has been conducted to find culture dependent and independent studies on *I*. *typographus* associated fungi using science search portals. First, we searched in the Web of Science (01/09/2025) with the specific termini “*Ips typographus* OR bark beetle AND fung* OR mycobiome OR Ophiosto*” which resulted in 28 relevant publications. Further searches in Google scholar and ResearchGate did not lead to additional findings. Furthermore, the bibliography of each publication found has been examined to detect further studies. Dissertations have been included in the survey (e.g., Kirschner, 1998), however, Bachelor and Master theses not. A total of 58 studies from 18 countries were evaluated to extract the fungal species that have been documented in association with *I. typographus* (Supplementary Table S1, Supplementary List S3 and Supplementary Figure S1). One study did sample in two geographic regions in Europe and therefore we included these as two data sets (Linnakoski et al., 2010).

**Figure 1:**
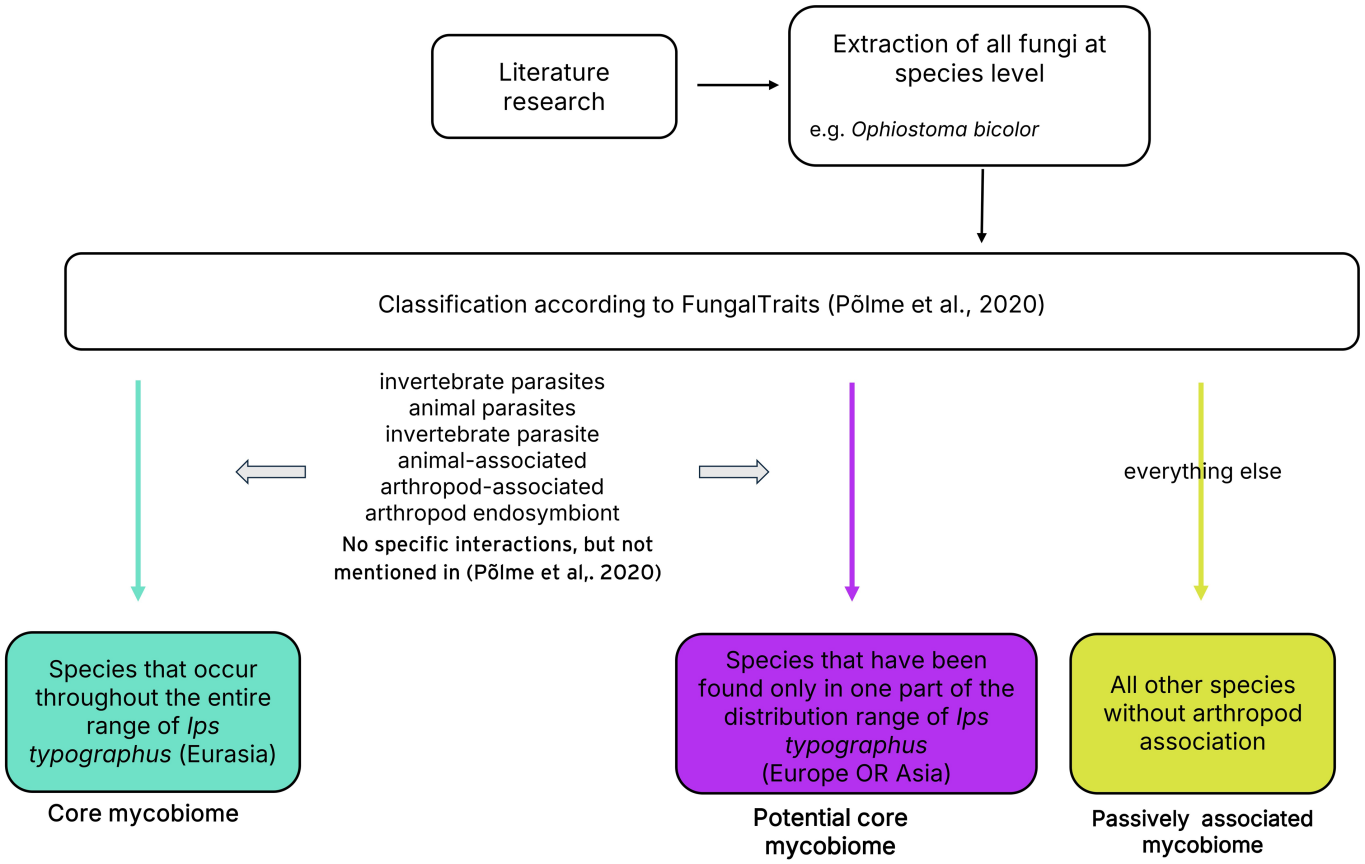
From literature research to categorization. Step-by-step description of the categorization of fungal species for the classification to the core mycobiome, potential core mycobiome, and passively associated species of *Ips typographus*.

Only fungal cultures determined at species level without cf. (confer, indicates that a specimen is similar to a known species but not definitively identified as that species) have been considered for the meta-analysis. The names of the fungal species are based on the ‘current names’ in the Index Fungorum nomenclatural database. With the exception of *Grosmannia penicillata*, which is listed as *Ceratocystis penicillata* in Index Fungorum, but is referred to as *G. penicillata* in the current literature and therefore also in our work (Zipfel et al., 2006). The categorization of fungal species into three distinct groups — namely, *core mycobiome*, *potential core mycobiome*, and *passively associated* — is predicated on the direct association between bark beetles and fungi, in addition to the geographic distribution (Figure 1). First, we made a list of all species that appeared in the literature. All these fungi were categorized based on FungalTraits which represents a comprehensive database compiling functional traits of fungi (Põlme et al., 2020) with the following exceptions: based on studies of Harrington, 1993 *Ceratocystiopsis* spp., were classified as pathogenic fungi instead of wood saprotrophs and *Cylindrobasidium ipidophilum* as arthropod-associated as mentioned by FungalTraits (Põlme et al., 2020). *Acaromyces ingoldii,* an invertebrate parasite on mites, were classified in the category of no specific interaction (Boekhout et al., 2003). After Kirschner et al. (2001) *Chionosphaera cuniculicola* was classified as arthropod associated.

Only species directly associated with arthropodes, such as endosymbiotic partners or parasites, were considered for the core and potential core mycobiome. All other taxa were classified as passively associated. To qualify as part of the core mycobiome, a species had to be detected at least once throughout the beetle’s geographic distribution range, in both Europe and Asia. Species found in only one geographic area were placed in the potential core list. This latter group highlights any biases present in current scientific literature, including geographic and methodological biases. For the two studies done in Russia, we employed the Ural Mountains, commonly regarded as the primary geographical boundary dividing Europe and Asia (e.g., Peel et al., 2007), to categorize them geographically. Consequently, these studies from the Karelia and Leningrad regions were geographically classified as data sets from Europe. All graphics were created using R packages ggplot2, VennDiagramm and treemap in R 4.3.0 (Dixon, 2003; Wickham, 2016; Tennekes, 2017; Chen and Boutros, 2011), Microsoft Powerpoint 2016 and Inkscape 1.0.2 and modified with Gimp 2.10.34. Rarefaction curves were created using iNext (Hsieh et al., 2016).

## Results

### Overview of studies reporting fungal associates

A total of 712 fungal species have been identified on *I*. *typographus* across 58 publications over the last 80 years (Supplementary Figure S1). Most of the studies were carried out in Europe (52), the majority of them in central (25) and northern Europe (20). Only six studies are from Asia (Japan and China) (Supplementary Figure S1). This local bias results in a corresponding taxonomic diversity with most of the fungal records (over 90% of the overall fungal diversity) coming from European studies with more than 50% of these records being from central Europe. Only 7.2% of the species were documented exclusively from Asian studies (Supplementary Figure S2).

Overall, the proportion of studies that have methodically worked exclusively on the cultivation of fungi (52) is predominant, and many studies have specifically focused on cultivating filamentous fungi (28) or even certain symbiotic taxa such as fungal pathogens as targets (6). Advanced genetic methods were used in six studies, namely metabarcoding on the ITS region in four, one metagenome sequencing and sequencing of fungal clone libraries in another one. Fungal species were cultivated in 54 studies (two studies here also used advanced genetic methods and are therefore listed under both methods).

**Figure 2:**
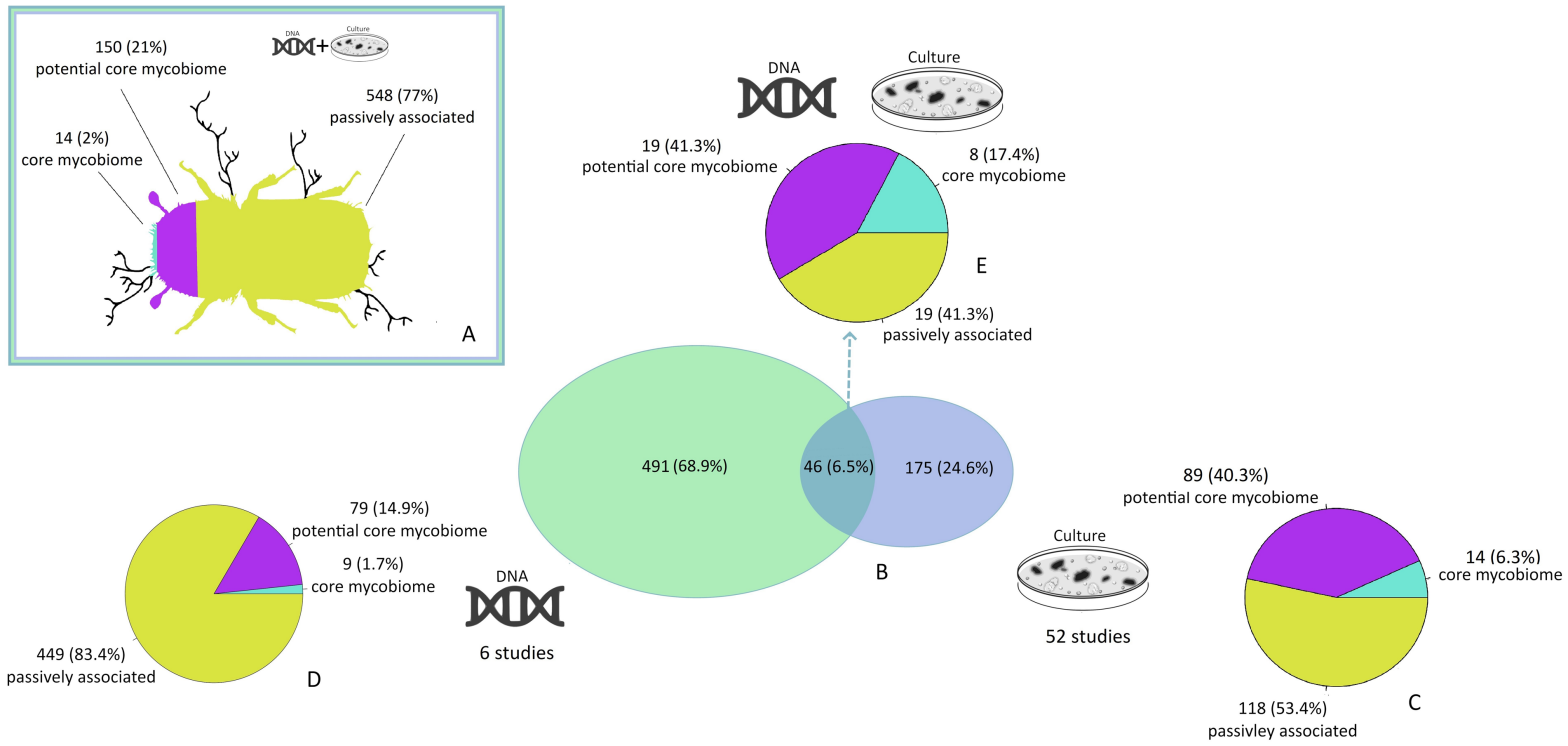
The classification of the mycobiome of *I. typographus*. **(A)** Proportions of the core mycobiome, potential core mycobiome, and passively distributed fungal species associated with *I. typographus*, compiled from all 58 studies using various detection methods. **(B)** Venn Diagram illustrating the fungal species identified by cultivation in culture (blue), those detected via next-generation sequencing (green), and species detected by both methods (dark green) in scientific literature. **(C)** Proportion of fungal species classifications based solely on cultivation methods from 52 studies. **(D)** Classification of fungal species identified through modern molecular methods across six studies. **(E)** Proportion of species classification detected concurrently by both cultivation and molecular methods.

**Figure 3:**
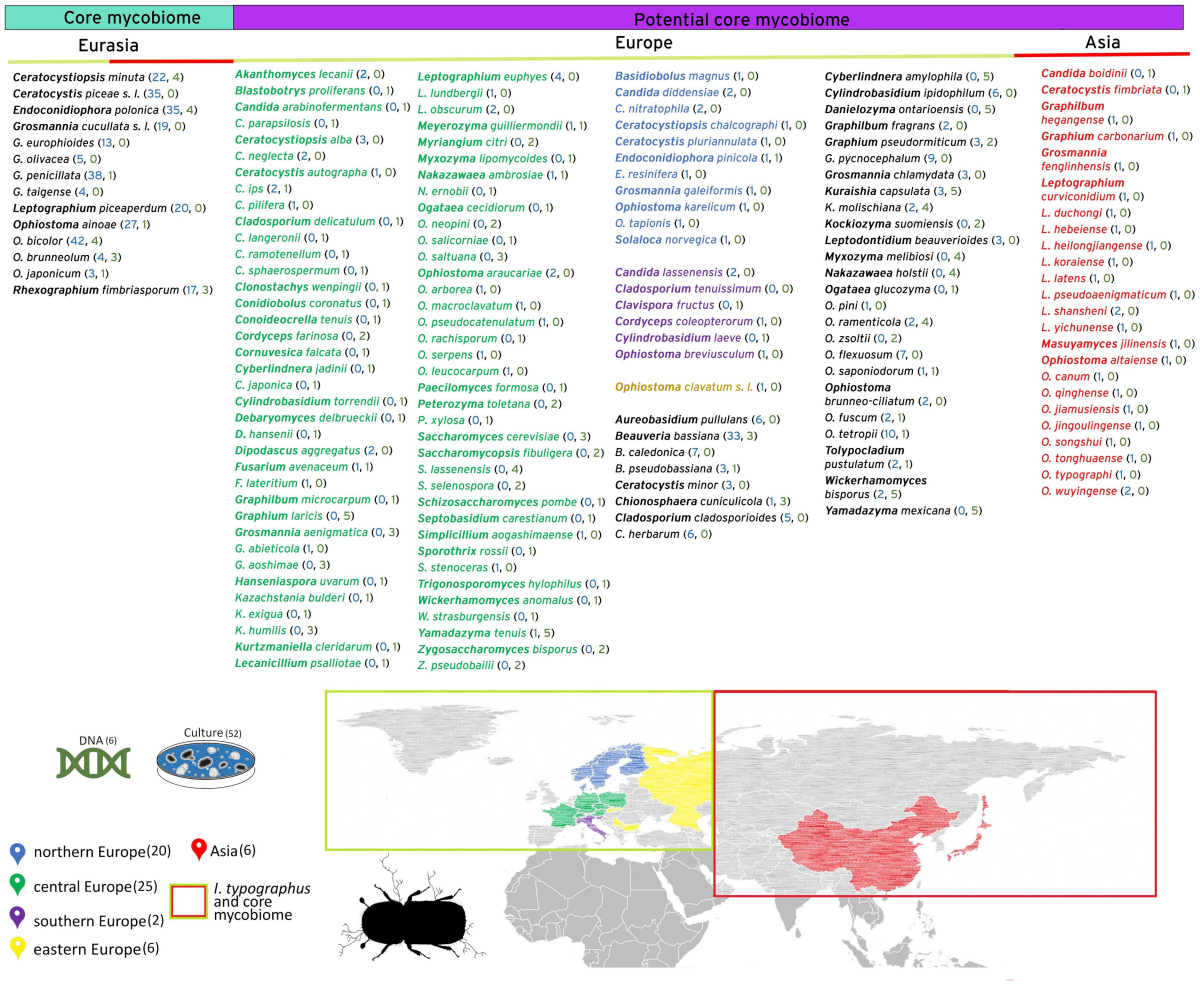
Arthropod-associated fungal species exhibiting specific geographic distribution patterns: The core mycobiome includes species identified in studies conducted in both Europe and Asia. The potential associated mycobiome consists of species documented in either Europe or Asia exclusively. Colors indicate the geographic origin of the species: Central Europe (green), Northern Europe (blue), Southern Europe (violet), and all European regions (black). Species from Asia are marked in red. Following each species, the numbers in parentheses represent the count of publications in which the species were reported, with molecular studies shown in green and cultivation studies in blue.

In 20 studies, pure cultures were identified through Sanger sequencing, while 34 studies relied solely on morphological characteristics. Out of the entire 712 species dataset, incorporating both detection methods, 14 species (2%) fall under the core, 150 (21%) under the potential core and the majority with 548 (77%) under the passively associated mycobiome (Figure 2). Cultivation methods, including morphological and Sanger sequencing, detected 175 species—constituting ~25% of all identified species and 89 potential core species (~50%). Modern sequencing techniques exclusively detected 69% of all recorded species but with the majority ~83% of these being classified as passively associated. Approximately 50% of all species classified as core or potential core have been detected using advanced molecular methods. There was an overlap of 46 fungal species (6.5%) identified by both cultivation and next-generation sequencing including all 14 core species (Figures 2, 3). From the species detected by both molecular and cultivation methods, ~59% were classified as core or potential core species and 19 species (~41%) as passively associated, among them *Alternaria alternata*, *Botrytis cinerea*, *Epicoccum nigrum*, and *Fusarium solani*. From the overall list of 548 fungal species classified as passively associated, 448 were detected only once, 87 appeared in 2–3 studies, nine in 4–5 studies and four in 6–8. The most frequently detected species include *Corinectria fuckeliana* and *Epicoccum nigrum* (six studies), *Alternaria alternata* (seven studies), and *Trichoderma viride* (eight studies).

### Taxonomic and functional classification of fungal associates from the literature

Almost 72.2% of all detected fungal species belong to the Ascomycota, which thus represent the dominant division. Another 22.9% are made up of species from the Basidiomycota. The remaining 4.9% are distributed among Chytridiomycota, Cryptomycota, Entomophthoromycota, Microsporidia, Mortierellomycota, Mucoromycota, Oomycota, Zoophagomycota and Zygomycota (Supplementary Figure S3 and Supplementary Table S2).

From the 712 identified fungal species, 14 species were recorded over the entire geographic distribution range of *I*. *typographus* and were categorized thereon as core mycobiome (2%) (Figure 3). All of them belong to the families of Ophiostomataceae and Ceratocystidiaceae, both containing predominantly arthropod-associated fungi. 150 *I*. *typographus* associated species were documented in only a part of its distribution range either Europe or Asia and were classified as the potential core mycobiome (21%) e. g. *O*. *wuyingense* has only been found in Asia so far (Chang et al., 2019). The potential or regional mycobiome includes positively associated bark beetle species e. g. from the genus *Ophiostoma*, but also endosymbiotic yeasts from the genus *Wickerhamomyces* and insect pathogens including *Acanthomyces lecanii* and *Beauveria bassiana*. At this scale, a differentiation between records of Asia, central-, northern, and southern Europe was found, as well as species that were recorded e. g. over all three European regions (Figure 3). However, the remaining 548 species were classified as passively associated species (77%) (Figure 2).

**Figure 4:**
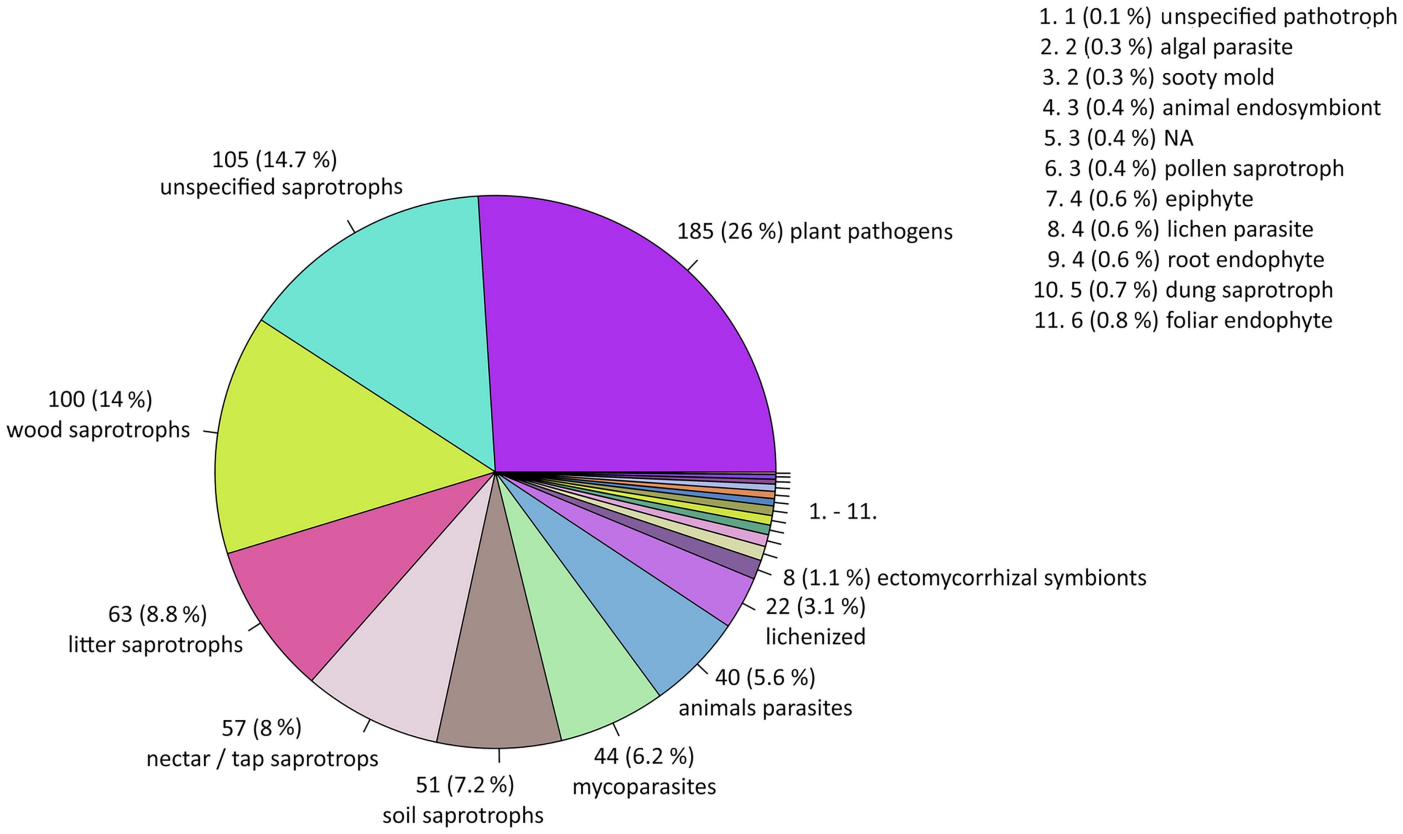
Fungal species represented by their type of trophy.

The highest number of reported species are saprotrophic fungi with 384 species (54%) and plant-parasitic fungi with 185 species (26%). Another approx. 5–6% each are mycoparasites and animal parasites. Animal endosymbionts (gut symbionts), a lichen, an ectomycorrhizal symbiont and an algal parasitic fungus were represented by single records (Figure 4, and further summarized in Supplementary Figure S4). The species-rarefaction curves demonstrate a pronounced surge in the number of passively associated species in comparison to the potential mycobiome and showing the core mycobiome to reach a point of saturation (Supplementary Figure S5). Furthermore, the majority of detected species, specifically the ones classified as passively associated, have been detected within the last four years (Supplementary Figure S6). For the 164 core and potential core species, a method bias was observed in the detection proportions between yeasts and filamentous fungi. The species record from exclusively cultivation-based studies was composed of approximately 86% filamentous fungi, whereas molecular studies primarily detected yeasts (including dimorphic yeasts), accounting for about 58% of their findings. Species identified by both methods exhibited an intermediate distribution, with roughly 33% yeasts and 67% filamentous fungi (Supplementary Figure S7).

## Discussion

This meta-analysis integrating 58 scientific studies offers a comprehensive exploration of the intricate association of *I. typographus* with its diverse fungal community across the beetle’s geographic distribution. Our study underscores the presence of a core mycobiome associated with *I*. *typographus*, characterized by 14 species that consistently appear throughout its range. It also highlights the existing bias of the fungal detection methods across the >80 years research history as well as a strong bias for sampling in European areas while largely neglecting Asia. This imbalance is reflected in the substantial potential core mycobiome, comprising 150 species identified in only one geographic region. It is plausible that further research will confirm some of these fungi as integral members of the core community. Nonetheless, the identification of a total of 712 fungal species illustrates that this beetle, rather than being an isolated ecological entity, is intricately linked to a highly diverse and complex fungal ecosystem (Six, 2012; Birkemoe et al., 2018).

### Ecological importance of the mycobiome

The fungal species classified as part of the core mycobiome in this meta-analysis belong predominantly to the Ophiostomataceae and Ceratocystidiaceae families which are actively dispersed by the beetles. Interestingly, all of these fungi are potent plant pathogens that extend into the phloem and wood of host trees, often leading to tree mortality in conjunction with beetle activity (Shi et al., 2022). This plant pathogenic function combined with positive effects on beetle nutrition make these fungi integral to the lifecycle of different bark beetles (Cale et al., 2016; Wingfield et al., 2017; Six and Elser, 2020). Additionally, fungi from these families, as well as those in the Microascaceae, can undermine tree defenses by degrading phenolic compounds (Hammerbacher et al., 2013; Barcoto and Rodrigues, 2022) and produce volatile attractant compounds that play a crucial role in beetle ecology, probably even aiding in aggregation (Kandasamy et al., 2019; Shi et al., 2022; Zhao et al., 2019). It has also been demonstrated that different bark beetle-associated fungi can complement each other by playing distinct roles in the degradation of plant-defense compounds (Zhao et al., 2019). The ability of the core mutualist *Endoconidiophora polonica* (previously *Ceratocystis polonica*) to degrade defensive phenolic compounds (Kandasamy et al., 2019) in combination with its phytopathogenic nature (Christiansen, 1985) exemplifies the ecological significance of fungal symbionts for host colonization.

Another crucial ecological group frequently detected alongside *I. typographus* includes symbiotic yeasts, such as *Kuraishia* spp., *Wickerhamomyces* spp., *Nakazawaea* spp., and *Ogataea* spp. These yeasts are highly prevalent in fungal communities throughout the beetle’s lifecycle (e.g., Baños-Quintana et al., 2024). Their detection has primarily resulted from recent next-generation sequencing studies (Supplementary Figure S7), which were predominantly conducted on samples from Europe, thus currently placing these species in the potential core community. Some of these yeasts have the genomic potential to significantly influence host beetle nutrition (Davis, 2015; Cheng et al., 2023) and some are likely also involved in intraspecific communication within *I. typographus* through pheromone production (Leufvén et al., 1984).

Despite this, a significant proportion (77%) of the fungal associates in our data are identified as passively associated species not showing a stable symbiosis with the beetle. Bark beetles play an important ecological role as vectors of plant pathogens and wood decomposers, strongly impacting the forest ecosystem by affecting tree health or wood decomposition (Masch et al., 2025; Seibold et al., 2022; Birkemoe et al., 2018). Therefore, many of these forest fungi can be found in the passively associated species list, among them prevalent deadwood saprotrophs, such as *Exidia glandulosa*, opportunistic pathogens like *Corinectria fuckeliana*, and key decomposers such as *Fomitopsis pinicola*, which colonize dead coniferous wood following bark beetle activity (Vogel et al., 2017). In fact, many wood-decaying fungi, such as *Stereum sanguinolentum* and *Fomitopsis pinicola*, were found in the data, which are pioneers colonizing fresh coniferous deadwood (Kleist, 2001; Gramss, 2020). But also species being encountered on more decomposed wood, such as *Postia sericeomollis* (Ruokolainen et al., 2018), are distributed by *I. typographus*. Also plant pathogens without a strong association with the beetles are vectored frequently, for example we see an irregular association of *I. typographus* with the aggressive pathogenic fungi *Corinectria fuckeliana* which has been detected in six studies and is infecting Norway spruce (Pettersson et al., 2018), as well as the white-rot fungus *Phellinus viticola* (Solheim, 1992b). Overall, no passively associated species were consistently found across the majority of studies, which could be influenced by the existing research foci in many culture-based studies. However, the most frequently identified species, *Alternaria alternata* and *Trichoderma viride*, were detected in eight and seven studies, respectively, encompassing both one molecular and otherwise culture-based methods. The absence of these species in the other molecular studies suggests that their infrequent detection is not caused by the methodological bias across the data set, but rather by a lack of strong association with the beetle. Importantly, as all fungi not classified as insect-associated were automatically moved into the passively associated list and given the complex ecologies of fungi, some of these may, in fact, have a closer association with the beetle than currently recognized.

Beyond generalist species such as *Alternaria alternata*, also fungi exhibiting high specialization could be detected among the fungal taxa (Grucmanová and Holuša, 2013). These were, for example, plant pathogens such as *Ophiostoma* spp., but also nematode-trapping fungal species (*Hohenbuehelia*, *Nematoctonus*, and *Orbilia*) which antagonize entomophilic or parasitic nematodes. The role of these fungi in maintaining gallery hygiene and protecting the beetle from antagonistic nematodes remains to be studied. In addition, *I. typographus* harbors fungal entomopathogens, including species from the genera *Beauveria*, *Ancanthomyces*, and *Isaria*, alongside diverse plant pathogens, wood decomposers, and mycoparasitic fungi, which likely act as antagonists to the beetle’s fungal mutualists.

This high overall diversity of fungal taxa and their wide range of ecological functions demonstrates the complexity and importance of the entire community associated with *I. typographus* in terms of its developmental and colonization success, as well as host-tree degradation and nutrient cycling within the entire forest ecosystem.

### Regional differentiation of the mycobiome

The published literature highlights significant geographical variations in fungal associations with *I. typographus*, predominantly focusing on Europe. Within Europe, most studies originate from the central and northern regions due to the distribution of the host tree, *Picea abies*. However, there is a pronounced geographical research bias (discussed further below). The metadata could indicate that the mycobiome may include species that are geographically restricted. While some of these taxa may merely be transient environmental entities, others could be intimate associates of the beetle, offering essential ecological functions and being locally adapted. The climatic and ecological differences between Asia and Europe, along with variations in host tree species (predominantly Norway spruce in Europe and amongst others Yezo spruce (*Picea jezoensis*) in Asia; (Furuta, 1989)), might impact the fungi through differences in, e.g., tree defense chemicals, temperature, or humidity. Additionally, the genetic adaptation of hosts to distinct environmental conditions can shape their colonizing microbial communities. It is worth mentioning that in some studies mainly from Japan the local *I. typographus* populations have been classified as subspecies *I. typographus japonicus* (e.g., Furuta, 1989; Yamaoka et al., 1997). While such effects by the environment or host adaptations are rather speculative at this point it is fact that many of the highly adapted fungal species are spread directly or indirectly by the beetles, for example by the formation of fruiting bodies in the tunnels (Linnakoski et al., 2010). As a result, geographical barriers could affect not only populations of beetles but also their adapted fungi. Insights from other bark beetle systems, particularly invasive ones, suggest that these beetles often associate with local microbial communities or that their carried microbiomes adapt to local conditions (Rassati et al., 2019; Taerum et al., 2013). Understanding the geographical and functional diversity of *I. typographus*-associated fungi could improve predictions of beetle population dynamics in different regions and environmental contexts.

### Challenges and opportunities

This study incorporates over 80 years of research, with one of our main objectives being to highlight existing research biases that affect our current understanding of the mycobiome associated with beetles. The meta-analysis incorporates studies based on traditional culturing methods as well as modern genetic techniques such as metabarcoding. There is a clear bias toward studies that rely exclusively on culture-dependent methods (*n* = 52), from which the majority identified the cultures morphologically (34), compared to Sanger sequencing (18). Next-generation sequencing methods are newer and still much more expensive which is the reason why we find only six recent publications in our data set applying these methods. These modern techniques have substantially increased the detection of fungal species across the data set over the past 4 years (Supplementary Figure S6). This is most pronounced in species with passive associations and, to a lesser extent, in potential core taxa, but not observed in core taxa, likely influenced by the existing research biases. Such methodological discrepancies underscore the need for methodological standardization and cross-validation to accurately and reliably chart fungal biodiversity.

Traditional culture-based methods are limited to a small fraction of ‘culturable’ microbes and are subject to growth requirements, as well as media and enrichment biases. Morphological identification can be incorrect and cannot easily be re-evaluated. Furthermore, many of the studies had a specific research focus (28 focused on filamentous fungi and six on fungal pathogens), which suggests that they were likely to use selective methods and discard any other diversity found. For example, we observed a strong research focus among Eastern European studies on species of the genus *Beauveria*. Modern techniques, such as amplicon sequencing, have been shown to capture microbial biodiversity much more effectively (Gupta et al., 2019). However, this method also has its caveats such as capturing non-viable cells and offering a lower resolution at the taxonomic level. It is sensitive to DNA extraction bias, copy number variations in rRNA genes and PCR amplification bias which can result in discrimination against certain taxa (Hugerth and Andersson, 2017; Keck et al., 2023). Latter is a particularly significant issue for the diverse fungal kingdom: for example, standard Internal Transcribed Spacer (ITS) primers discriminate against certain Ophiostomatales fungi, including many bark and ambrosia beetle associates, which is the reason for the additional usage of other fungal regions such as the Small Subunit ribosomal RNA (SSU) and Large Subunit ribosomal RNA (LSU) in these systems (Ibarra-Juarez et al., 2020; Diehl et al., 2022). For fungal communities, combining both methods is thought to improve accuracy and comprehensiveness of identification (Rieker et al., 2024). This has also been demonstrated in *I. typographus*, where applying the two methods to the same samples produced different taxonomic results, indicating that the two methods complement each other (Giordano et al., 2013; Veselská et al., 2023). The meta-analysis presented here suggests the same, indicating that a combination of both methods is preferable for getting a broad diversity. We see that the purely culture-dependent studies do not report many potential core species such as yeasts (Supplementary Figure S7), whereas molecular studies detect a large amount of likely non-associated taxa (Figure 3 and Supplementary Figure S6). However, this picture may change with a greater number of next-generation sequencing studies. In addition to these technical biases, the results show that 77% of fungal detections are passively associated and therefore rather random for this well-known model species. If we transfer this to highly complex systems such as soil or dead wood, a crucial question emerges: How can we distinguish valuable data from random occurrences in order to identify genuine interactions? To understand the relationships between organisms and their associated symbionts, intensive field surveys with standardized protocols and controlled experiments would be helpful (e.g., Hulcr et al., 2020; Baños-Quintana et al., 2024).

In addition to the methodological bias, a pronounced geographic research bias persists. The overall number of publications on the Asian distribution of beetles is, with altogether six publications, very small (Supplementary Figure S2). Small is also the range of methodologies used to analyze these Asian communities. In fact, only one study has used modern culture-independent methods (Liu et al., 2025). Therefore, it is important to note that we can be confident of a species presence, but not of their absence. For example, the finding that yeasts actually dominate *I. typographus* fungal communities is a relatively recent discovery made possible by culture-independent methods. As for example yeasts are underrepresented or absent in studies of the Asian distribution, potentially present yeasts may simply not have been the target organisms, or may not have been detected due to different cultivation or primer requirements. Therefore, we cannot speak of a local fungal community, but rather of a potential core community, as the depth of the data does not allow us to conclude that species are truly absent. With a higher representation of studies from Asia, we can expect substantial changes especially in the potential core community likely shifting multiple species either into the core or the passively associated community.

While this meta-analysis demonstrates the complexity of the fungal community, it is important to note that the entire biotic interaction network comprises many other significant organisms, including bacteria, nematodes, archaea, protozoa, mites, and viruses, all of which play a role in interacting with the beetle, tree, and fungal associates. More comprehensive research is needed, integrating standardized protocols and advanced genomic tools to study multiple organismic levels, as well as ecological modeling, in order to elucidate the dynamic feedbacks within the bark beetle interaction network. The development of databases capable of adequately recording and reporting the complexity of the interactions observed among bark beetles and fungi, or indeed any members of the close biotic network, in a standardized manner would be highly advantageous for fundamental as well as applied research (see an inspiring example for bees here: https://beebiome.org/; Engel et al., 2016).

## Conclusion

Our review identified 712 fungal species associated with *I. typographus,* indicating that it is integrated into a complex fungal ecosystem rather than functioning as an isolated entity. This meta-analysis revealed diverse fungal communities, including core, potential core and passively associated mycobiomes. This diversity suggests that the beetle’s ecological and evolutionary success, along with its tree-killing potential, may be profoundly shaped by its symbiotic relationships with fungi that aid in overcoming host defenses, enhancing nutrient acquisition and affecting behavior, or act as competitive antagonists. These findings emphasize the critical need for interdisciplinary approaches to analyzing forest ecosystems, taking into account the foundational role of fungal symbionts in the ecology of bark beetles. Despite remarkable advancements in our understanding of fungal associates, substantial gaps persist concerning methodological and geographical biases, and the ecological roles and dependencies of many fungi in relation to their beetle hosts remain unclear. While our study provides an overview of the current research landscape, it is evident that the mycobiome landscape will continue to evolve with future studies. Furthermore, interactions between beetles and fungi are likely to be affected by environmental conditions such as temperature and humidity, which are shifting rapidly due to climate change. A comprehensive understanding of the adaptive mechanisms within tri-trophic interactions involving host plants, fungi and insects is required to understand, predict and manage current and future spruce bark beetle outbreaks.

## Data Availability

The datasets presented in this study can be found in online repositories. The names of the repository/repositories and accession number(s) can be found in the article/Supplementary material.

## Supplementary material

SUPPLEMENTARY TABLE S1 Overview and information about the studies used for this meta-analysis.

SUPPLEMENTARY TABLE S2 Meta-data of all fungal species, their classification, geographic distribution and detailed method information used for the analysis.

SUPPLEMENTARY List S3 Meta-data of all references.

SUPPLEMENTARY FIGURE S1 Total number of species in publications from different regions in Europe and Asia. The study by Linnakoski et al. (2010) was evaluated twice, since it analyzed samples from both Northern and Eastern Europe.

SUPPLEMENTARY FIGURE S2 Taxa separated by their distribution based on the 58 publications.

SUPPLEMENTARY FIGURE S3 Affiliation of the detected species to a fungal phyla.

SUPPLEMENTARY FIGURE S4 Summary of the main nutritional types of fungal species, based on Figure 4.

SUPPLEMENTARY FIGURE S5 Species accumulation curve of the species classified in the three main associated groups.

SUPPLEMENTARY FIGURE S6 Abundance of the species appearing in the scientific literature over the last 80 years, classified within the three main associated groups.

SUPPLEMENTARY FIGURE S7 Recovery of yeasts vs. filamentous fungi across the two identification methods. The core and potential core taxa, according to the methodology used (DNA: blue; cultivation: green), are shown in a Venn diagram. Pie charts show the respective proportions of species growing as yeasts (including dimorphic yeasts) and filamentous species.
