# Supplementary figures and images for: Fungal backpackers—the mycobiome of Ips typographus after more than 80 years of research
Source: Front Microbiol. 2026 Jan 21;16:1695278. doi: 10.3389/fmicb.2025.1695278 (PMC12870000; doi:10.3389/fmicb.2025.1695278)

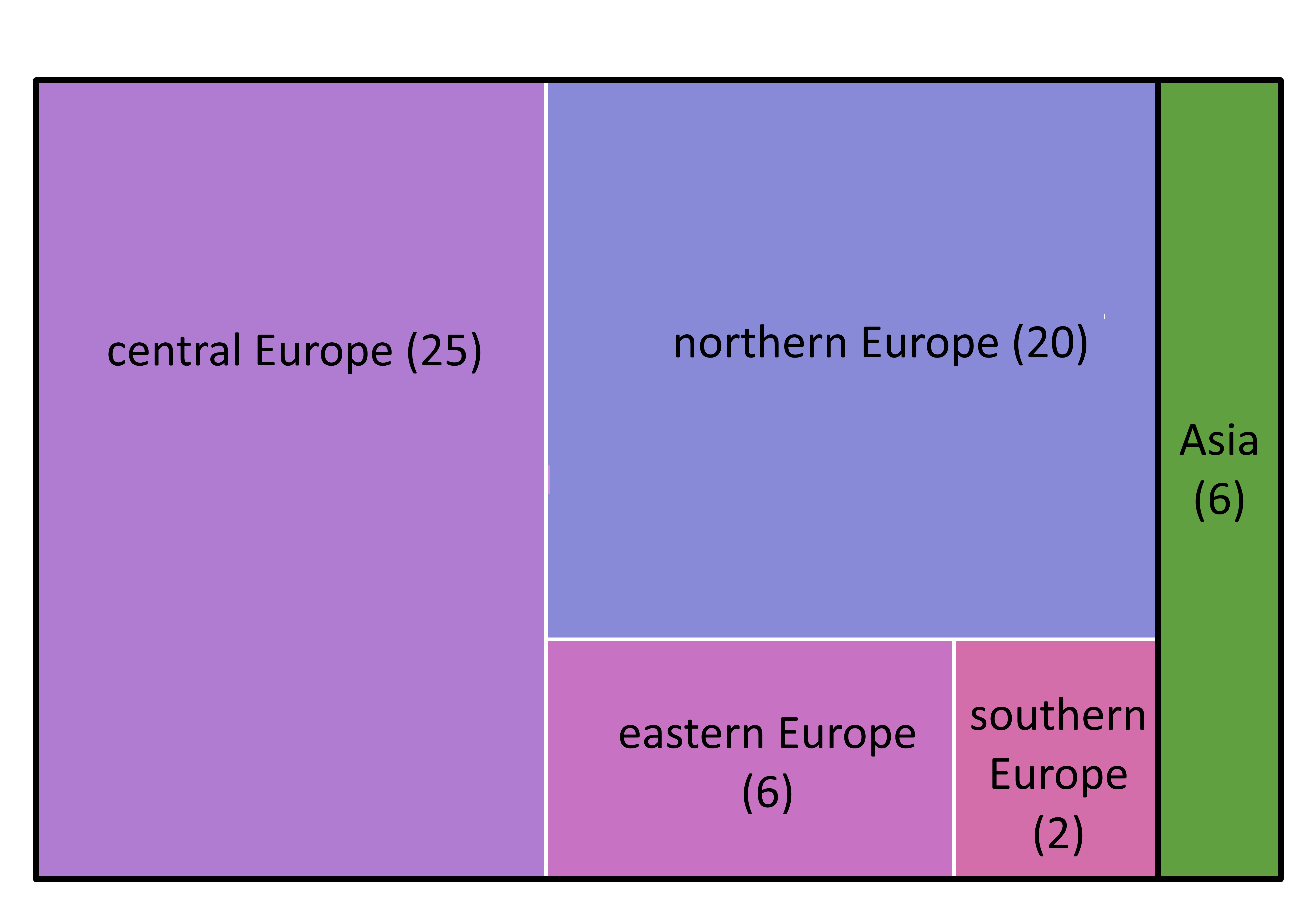

Supplement: Supplementary file 1 [file Image_1.jpeg]

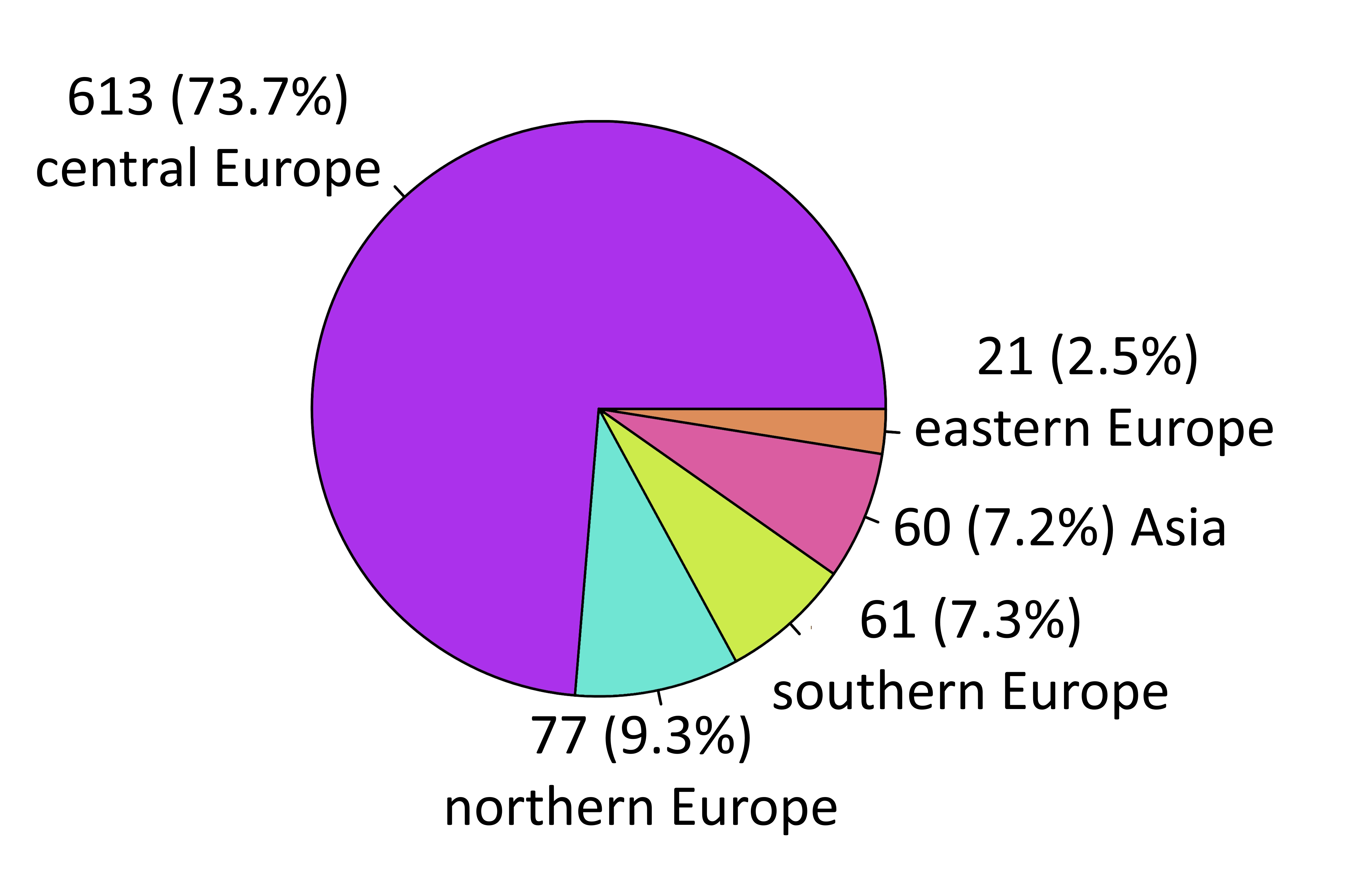

Supplement: Supplementary file 2 [file Image_2.jpeg]

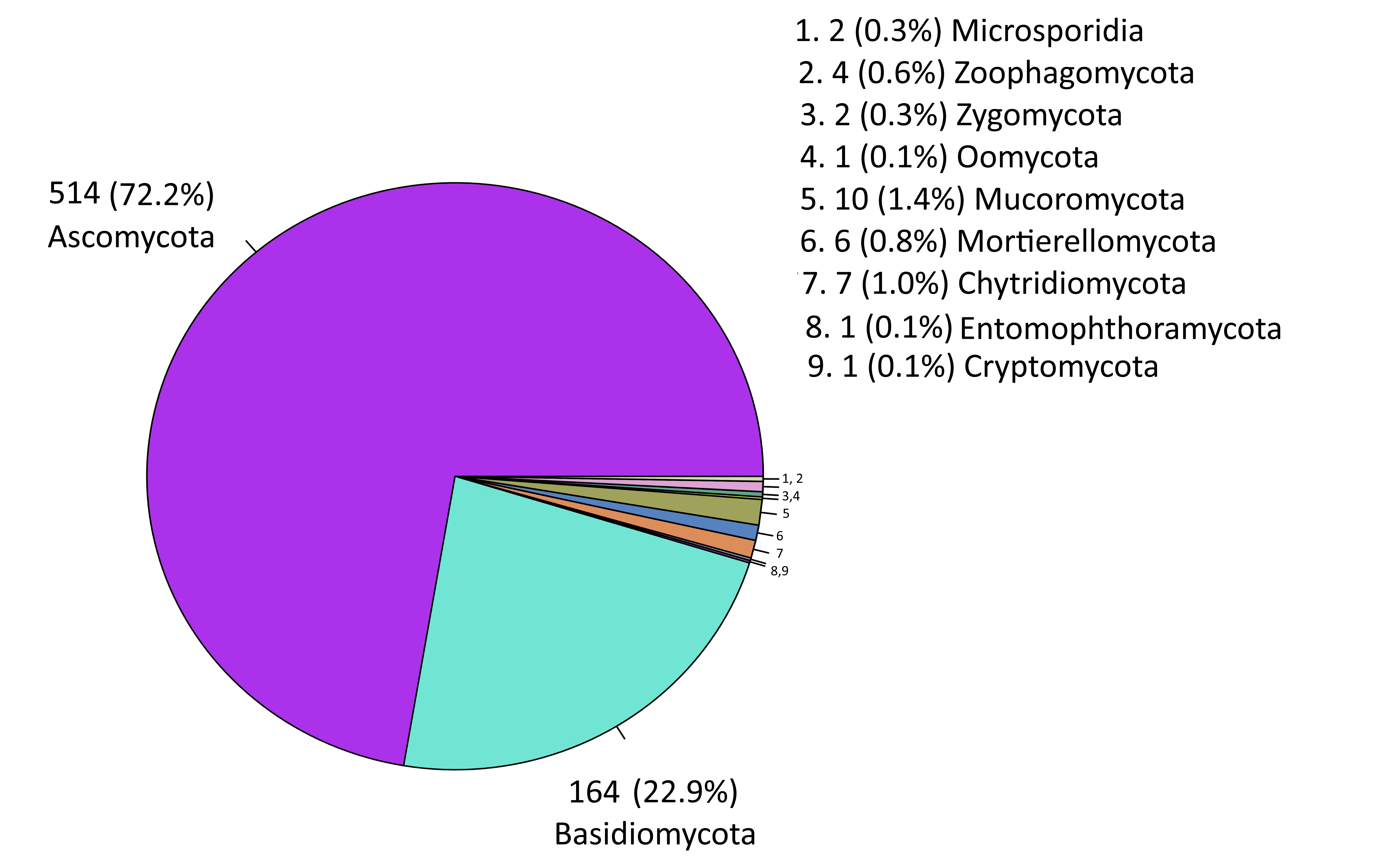

Supplement: Supplementary file 3 [file Image_3.jpeg]

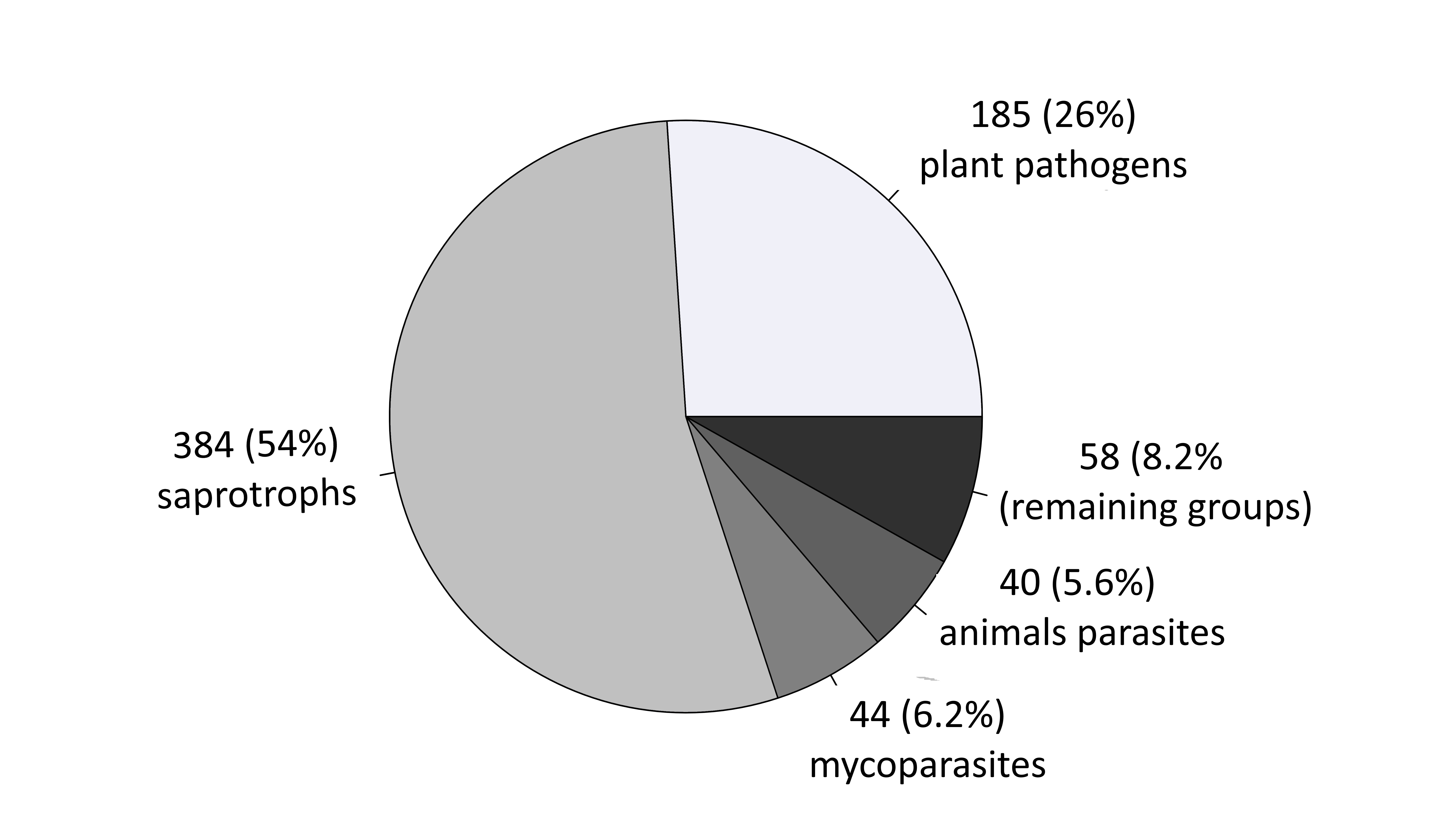

Supplement: Supplementary file 4 [file Image_4.jpeg]

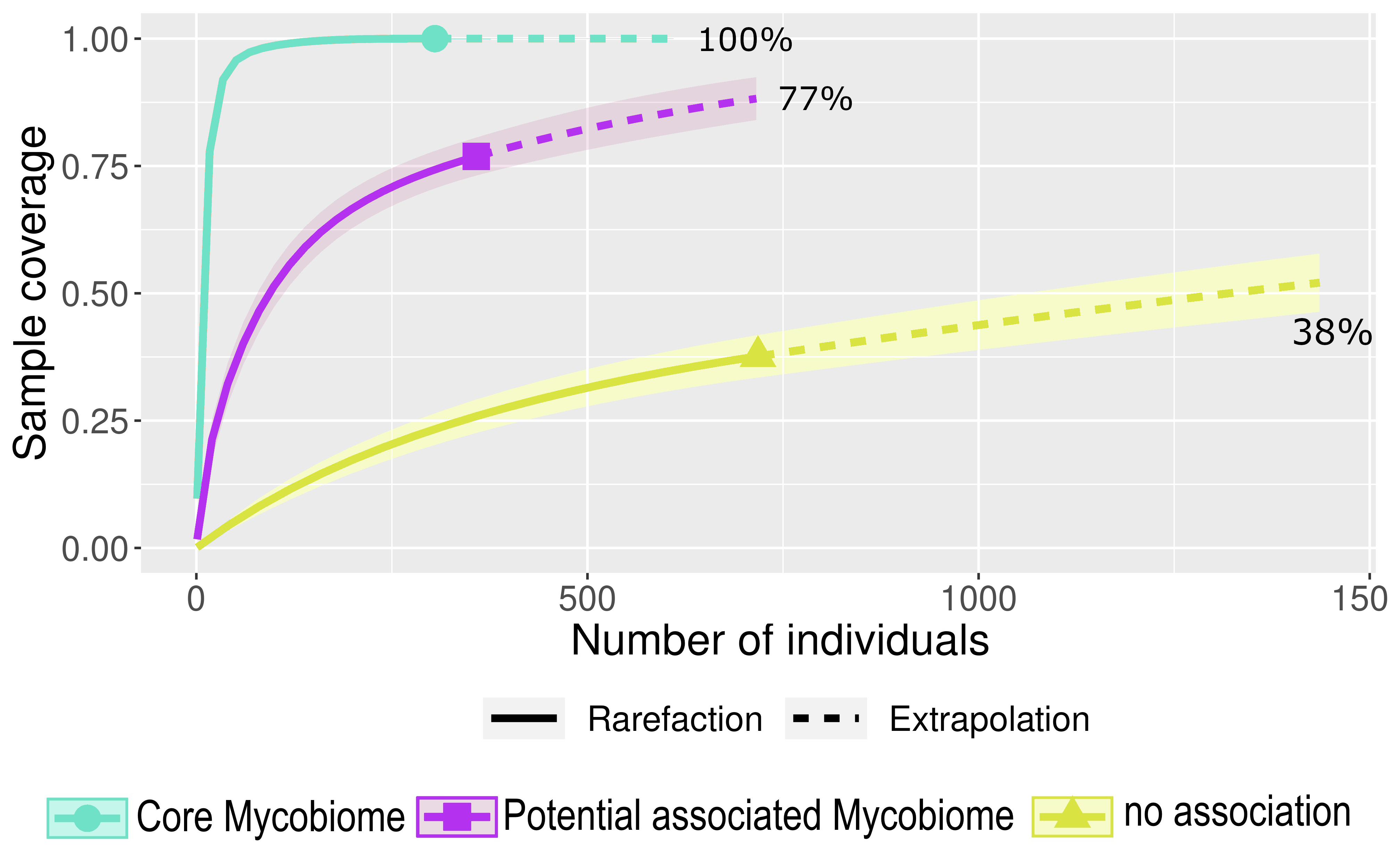

Supplement: Supplementary file 5 [file Image_5.jpeg]

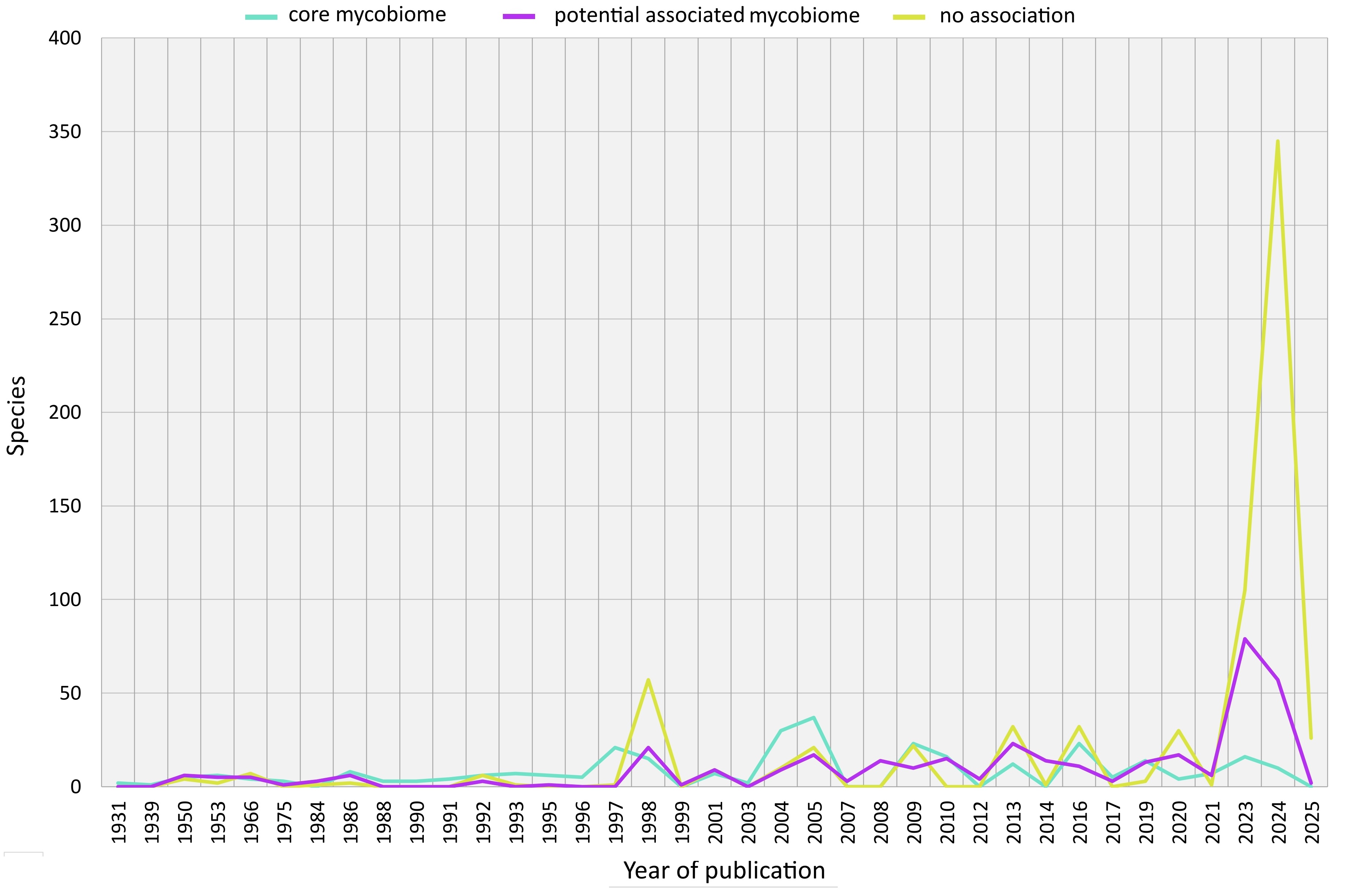

Supplement: Supplementary file 6 [file Image_6.jpeg]

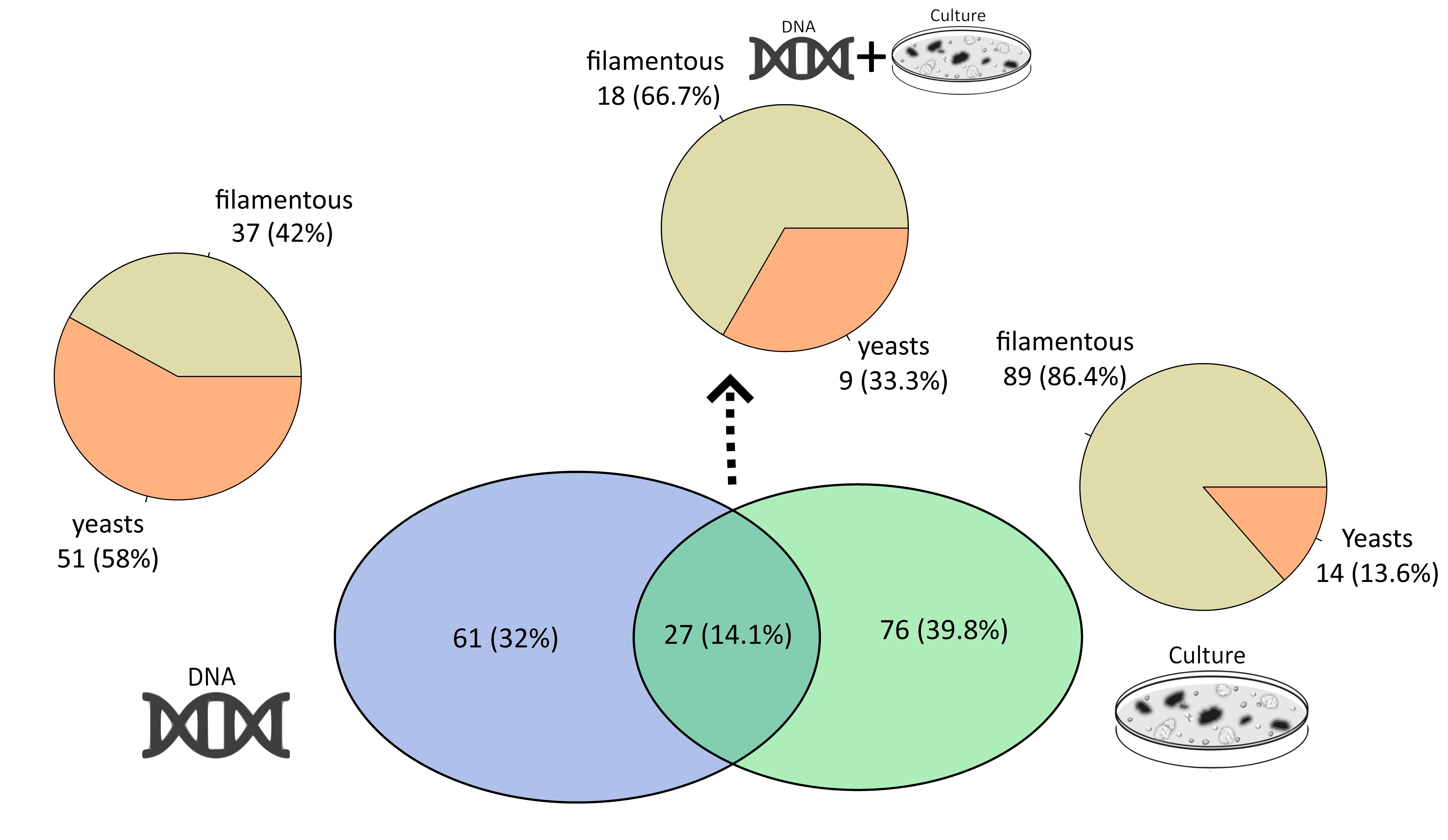

Supplement: Supplementary file 7 [file Image_7.jpg]
