# Supplementary material for: Fungal backpackers—the mycobiome of Ips typographus after more than 80 years of research
Source: Front Microbiol. 2026 Jan 21;16:1695278. doi: 10.3389/fmicb.2025.1695278 (PMC12870000; doi:10.3389/fmicb.2025.1695278)
Supplement: Supplementary file 10 [file Table_3.docx]

**References used for the Review**

Baños-Quintana, A. P., Gershenzon, J. and Kaltenpoth, M. (2024). The Eurasian spruce bark beetle *Ips typographus* shapes the microbial communities of its offspring and the gallery environment. Frontiers in Microbiology, 15, p.1367127.

Barta, M., Kautmanová, I., Čičková, H., Ferenčík, J., Florián, Š., Novotný, J. and Kozánek, M. (2018). Hypocrealean fungi associated with populations of Ips typographus in West Carpathians and selection of local Beauveria strains for effective bark beetle control. *Biologia*, *73*(1), pp.53-65.

Barta, M., Takov, D., Pilarska, D., Doychev, D., Horáková, M. K. (2020). Entomopathogenic fungi of the genus Beauveria and their pathogenicity to *Ips typographus* (Coleoptera: Curculionidae) in the Vitosha National Park, Bulgaria. Journal of Forest Science, 66(10), 420.

Chakraborty, A., Purohit, A., Khara, A., Modlinger, R., Roy, A. (2023). Life-stage and geographic location determine the microbial assemblage in Eurasian spruce bark beetle, *Ips typographus* L. (Coleoptera: Curculionidae). Front. Forests Global Change, 6(1176160): 10-3389.

Chakraborty, A., Modlinger, R., Ashraf, M. Z., Synek, J., Schlyter, F., Roy, A. (2020). Core mycobiome and their ecological relevance in the gut of five *Ips* bark beetles (Coleoptera: Curculionidae: Scolytinae). Frontiers in Microbiology, 11, p.568853.

Chang, R., Duong, T. A., Taerum, S. J., Wingfield, M. J., Zhou, X., Yin, M., de Beer, Z. W. (2019). Ophiostomatoid fungi associated with the spruce bark beetle *Ips typographus*, including 11 new species from China. Persoonia. 42 50-74. doi:10.3767/persoonia.2019.42.03. PMID: 31551614; PMCID: PMC6712535.

Furniss, M. M., Solheim, H., Christiansen, E. (1990). Transmission of blue-stain fungi by *Ips typographus* (Coleoptera: Scolytidae) in Norway spruce. Annals of the Entomological Society of America, 83(4), 712-716.

Giordano, L., Garbelotto, M., Nicolotti, G., Gonthier, P. (2013). Characterization of fungal communities associated with the bark beetle *Ips typographus* varies depending on detection method, location, and beetle population levels. *Mycological Progress, 12, 127-140.*

Grosmann, H. (1931). Beitrage zur Kenntnis der Lebensgemeinschaft zwischen Borkenkäfern und Pilzen. *Z. ParasitKde* 3, 56–102.

Hyblerová, S., Medo, J. and Barta, M. (2021). Diversity and prevalence of entomopathogenic fungi (Ascomycota, Hypocreales) in epidemic populations of bark beetles (Coleoptera, Scolytinae) in spruce forests of the Tatra National Park in Slovakia. *Annals of Forest Research*, *64*(1), pp.129-145.

Jacobs, K., Kirisits, T., Wingfield, M. J. (2003). Taxonomic re-evaluation of three related species of Graphium, based on morphology, ecology and phylogeny. Mycologia, 95(4), 714-727.

Jankowiak, R. (2004). Fungi associated with the beetles of *Ips typographus* on Norway spruce in Southern Poland. Acta Mycologica, 39(1), 105-116.

Jankowiak, R. (2004). Ophiostomatoid fungi associated with the spruce bark beetle (*Ips typographus*) new for Poland: occurrence and morphology. Phytopathol Pol, 33, 5-21.

Jankowiak, R. (2005). Fungi associated with *Ips typographus* on *Picea abies* in southern Poland and their succession into the phloem and sapwood of beetle‐infested trees and logs. Forest Pathology, 35(1), 37-55.

Jankowiak, R. and Hilszczanski, J. (2005). Ophiostomatoid fungi associated with *Ips typographus* [L.] on *Picea abies* [L.] H. Karst.] and Pinus sylvestris L. in North-Eastern Poland. Acta Societatis Botanicorum Poloniae, 74(4).

Jankowiak, R., Kacprzyk, M., Młynarczyk, M. (2009). Diversity of ophiostomatoid fungi associated with bark beetles (Coleoptera: Scolytidae) colonizing branches of Norway spruce (*Picea abies*) in southern Poland. Biologia, 64(6), 1170-1177.

Jankowiak, R., Strzałka, B., Bilański, P., Kacprzyk, M., Lukášová, K., Linnakoski, R., et al., (2017). Diversity of Ophiostomatales species associated with conifer-infesting beetles in the Western Carpathians. European Journal of Forest Research, 136, 939-956.

Käärik, A. (1975). Succession of Microorganisms during Wood Decay. In: Liese, W. (eds) Biological Transformation of Wood by Microorganisms. Springer, Berlin, Heidelberg. https://doi.org/10.1007/978-3-642-85778-2_4

Kirschner, R. (1998). *Diversität mit Borkenkäfern assoziierter filamentöser Mikropilze*. Dissertation.

Kirschner, R., & Oberwinkler, F. (1999). A new Ophiostoma species associated with bark beetles infesting Norway spruce. Canadian Journal of Botany, 77(2), 247-252.

Kirschner, R. (2001). Diversity of filamentous fungi in bark beetle galleries in central Europe. In Trichomycetes and other fungal groups (pp. 175-196). CRC Press.

Kirstis, T., Grubelnik, R., Führer E. (1999). Die ökologische Bedeutung von Bläuepilzen für rindenbrütende Borkenkäfer. In: Mariabrunner Waldbautage 1999 - Umbau sekundärer Nadelwälder / Hrsg. Von F. Müller. FBVA-Berichte; Schriftenreihe der Forstlichen Bundesversuchsanstalt Wien, 2000, Nr. 111, 237 S.

Kirisits, T. (2005). *Gloeocystidium ipidophilum*: Ein ungewöhnlicher Basidiomycete, der mit dem Buchdrucker assoziiert ist. Forstschutz Aktuell, 34, 14-17.

Kirisits, T. (2010). Fungi isolated from *Picea abies* infested by the bark beetle *Ips typographus* in the Białowieża forest in north‐eastern Poland. Forest Pathology, 40(2), 100-110.

Kotynkova-Sychrova, E., (1966). Mykoflora chodeb kurovcu v Ceskoslovensku. (The myco- flora of bark-beetle galleries in Czechoslovakia) Ceska Mykol. 20, 45–53.

Kowalski, T., Sowa, J. and Łakomy, P. (2019). Mykobiota w obrębie strzał zamierających świerków w Leśnym Kompleksie Promocyjnym" Puszcza Białowieska" i jej ekologiczne funkcje. *sylwan*, *163*(06), pp.496-507.

Krokene, P., Solheim, H., (1998). Pathogenicity of four blue-stain fungi associated with aggressive and nonaggressive bark beetles. Phytopathology 88: 39–44. <https://doi.org/10.1094/PHYTO.1998.88.1.39>

Lednev, G. R., Levchenko, M. V., Kazarstev, I. A. (2019). Mycobiota associated with the European spruce bark beetle (*Ips typographus*) in Leningrad Region. Mycology and Phytopathology 53(2):80-89.

Leufvén A., Bergström, G., Falsen, E. (1984). Interconversion of verbenols and verbenone by identified yeasts isolated from the spruce bark beetle *Ips typographus*. J Chem Ecol., 10(9):1349-61. doi: 10.1007/BF00988116. PMID: 24317586.

Leufvén, A., and Nehls, L. (1986). Quantification of different yeasts associated with the bark beetle, *Ips typographus*, during its attack on a spruce tree. Microbial ecology, 12, 237-243.

Linnakoski, R., De Beer, Z. W., Ahtiainen, J., Sidorov, E., Niemelä, P., Pappinen, A., Wingfield, M. J. (2010). Ophiostoma spp. associated with pine-and spruce-infesting bark beetles in Finland and Russia. Persoonia-Molecular Phylogeny and Evolution of Fungi, 25(1), 72-93.

Linnakoski, R., Mahilainen, S., Harrington, A., Vanhanen, H., Eriksson, M., Mehtätalo, L., ... & Wingfield, M. J. (2016). Seasonal succession of fungi associated with *Ips typographus* beetles and their phoretic mites in an outbreak region of Finland. PLoS One, 11(5), e0155622.

Linnakoski, R., Lasarov, I., Veteli, P., Tikkanen, O. P., Viiri, H., Jyske, T. et al., (2021). Filamentous fungi and yeasts associated with mites phoretic on *Ips typographus* in Eastern Finland. Forests, 12(6), 743.

Liu, C., Wang, H., Wang, Z., Liang, L., Li, Y., Liu, D. and Lu, Q. (2025). Distinct assembly processes of intestinal and non-intestinal microbes of bark beetles from clues of metagenomic insights. *Scientific Reports*, *15*(1), p.7910.

Mathiesen, A. (1950). Über einige mit Borkenkäfern assoziierte Bläuepilze in Schweden. *Oikos*, 275-308.

Mathiesen-Käärik, A. (1953). Eine Übersicht über die gewöhnlichsten mit Borkenkäfern assoziierten Bläuepilze in Schweden und einige für Schweden neue Bläuepilze. Statens skogsforskningsinstitut 43:4. <https://res.slu.se/id/publ/125112>

Milosavljević, M., Tabaković-Tošić, M., Radulović, Z., Marković, M. and Rindoš, M. (2021). Isolation, identification and phylogenetic position of entomopathogenic fungus Beauveria bassiana from Ips typographus in Serbia. *Fresenius Environ. Bull*, *30*, pp.9443-9448.

Moser, J. C., Perry, T. J., Furuta, K. (1997). Phoretic mites and their hyperphoretic fungi associated with flying *Ips typographus japonicus* Niijima (Col., Scolytidae) in Japan. Journal of Applied Entomology 121.1‐5: 425-428.

Novotný, D. and Jankovský, L. (2005). Notes on mycobiota associated with *Ips typographus* from the Šumava Mts. (Czech Republic). Czech Mycol. 57: 91-96.

Paciura, D., Zhou, X. D., De Beer, Z. W., Jacobs, K., Ye, H., & Wingfield, M. J. (2010). Characterisation of synnematous bark beetle-associated fungi from China, including *Graphium carbonarium* sp. nov. Fungal Diversity, 40, 75-88.

Persson, Y., Vasaitis, R., Langstrom, B., Öhrn, P., Ihrmark, K., Stenlid, J. (2009). Fungi Vectored by the Bark Beetle *Ips typographus* Following Hibernation Under the Bark of Standing Trees and in the Forest Litter. Microbial ecology. 58. 651-9. 10.1007/s00248-009-9520-1.

Reid, J., Iranpour, M., Rudski, S. M., Loewen, P. C., Hausner, G. (2010). A new conifer-inhabiting species of Ceratocystis from Norway. *Botany*, *88*(11), 971-983.

Rennerfeit, E. (1950). Über den Zusammenhang zwischen dem Verblauen des Holzes und den Insekten. *Oikos*, 2 : 120 – 137 .

Repe, A., Kirisits, T., Piškur, B., De Groot, M., Kump, B., Jurc, M. (2013). Ophiostomatoid fungi associated with three spruce-infesting bark beetles in Slovenia. *Annals of Forest Science*, *70*, 717-727.

Sallé, A., Monclus, R., Yart, A., Garcia, J., Romary, P., Lieutier, F. (2005). Fungal flora associated with *Ips typographus*: frequency, virulence, and ability to stimulate the host defence reaction in relation to insect population levels. Canadian journal of forest research, 35(2), 365-373.

Siemaszko, W. (1939). Fungi associated with bark beetles in Poland. Planta Polonica. 7(3):1-54.

Solheim, H. (1986). Species of Ophiostomataceae isolated from *Picea abies* infested by the bark beetle *Ips typographus*. *Nord. J. Bot.*, 6: 199 – 207.

Solheim, H. (1988). *Pathogenicity of some Ips typographus-associated blue-stain fungi to Norway spruce* (Vol. 40, No. 14, pp. 11-pp). Norsk institutt for skogforskning.

Solheim, H. (1991). Oxygen deficiency and spruce resin inhibition of growth of blue stain fungi associated with *Ips typographus*. Mycological Research, 95(12), 1387-1392.

Solheim, H. (1992a). The early stages of fungal invasion in Norway spruce infested by the bark beetle *Ips typographus*. Can. J. Bot., 70: 1–5.

Solheim, H. (1992b). Fungal succession in sapwood of Norway spruce infested by the bark beetle *Ips typographus*. Eur. J. For Path., 22: 136–148. <https://doi.org/10.1111/j.1439-0329.1992.tb01440.x>

Solheim, H. (1993). Fungi associated with the spruce bark beetle *Ips typographus* in an endemic area in Norway. Scandinavian Journal of Forest Research, 8(1-4), 118-122.

Veselská, T., Švec, K., Kostovčík, M., Peral-Aranega, E., Garcia-Fraile, P., Křížková, B., Havlíček, V., Saati-Santamaría, Z. and Kolařík, M. (2023). Proportions of taxa belonging to the gut core microbiome change throughout the life cycle and season of the bark beetle *Ips typographus*. FEMS Microbiology Ecology, 99(8), p.fiad072.

Viiri, H. and Weissenberg, K. V. (1995). Ophiostoma blue-staining fungi associated with *Ips typographus* in Finland. Aktuelt fra Skogforsk, 4: 58-60.

Viiri, H. (1997). Fungal associates of the spruce bark beetle *Ips typographus* L. (Col. Scolytidae) in relation to different trapping methods. Journal of Applied Entomology, 121(1‐5): 529-533.

Viiri, H. and Lieutier, F. (2004). Ophiostomatoid fungi associated with the spruce bark beetle, *Ips typographus*, in three areas in France. Annals of forest science, 61(3): 215-219.

Wang, Z., Liang, L., Wang, H., Decock, C., Lu, Q. (2024). Ophiostomatoid fungi associated with Ips bark beetles in China. Fungal Diversity 129, 283–364. <https://doi.org/10.1007/s13225-024-00546-7>

Wegensteiner, R., Stradner, A., Händel, U. (2014). Occurrence of pathogens in *Ips typographus* (Coleoptera: Curculionidae) and in other spruce bark beetles from the wilderness reserve Dürrenstein (Lower Austria). Biologia 69: 92–100. <https://doi.org/10.2478/s11756-013-0286-z>

Wegensteiner, R., Weiser, J. (2004). Annual variation of pathogen occurrence and pathogen prevalence in *Ips typographus* (Coleoptera, Scolytidae) from the BOKU University Forest Demonstration Centre. J. Pest. Sci. 77: 221–228.

Wegensteiner, R., Wermelinger, B., Herrmann, M. (2015). Chapter 7 - Natural Enemies of Bark Beetles: Predators, Parasitoids, Pathogens, and Nematodes, in: Vega, F.E., Hofstetter, R.W. (Eds.), Bark Beetles. Academic Press, San Diego, pp. 247–304. <https://doi.org/10.1016/B978-0-12-417156-5.00007-1>

Yamaoka, Y., Wingfield, M. J., Takahashi, I., Solheim, H. (1997). Ophiostomatoid fungi associated with the spruce bark beetle *Ips typographus* f. *japonicus* in Japan. *Mycological research*, *101*(10): 1215-1227. <https://doi.org/10.1017/S0953756297003924>

|  |
| --- |
|  |
|  |
|  |
|  |
|  |
|  |
|  |
|  |
|  |
|  |
|  |
|  |
|  |
|  |
|  |
|  |
|  |
|  |
|  |
|  |
|  |
|  |
|  |
|  |
|  |
|  |
|  |
|  |
|  |
|  |
|  |
|  |
|  |
|  |
|  |
|  |
|  |
|  |
|  |
|  |
|  |
|  |
|  |
|  |
|  |
|  |
|  |
|  |
|  |
|  |
| Veselska et al. 2023 |
| Viiri & Lieutier 2004 |
| Viiri 1997 |
| Virii & Weissenberg 1995 |
| Wang et al. 2024 |
| Wegensteiner et al. 2015 |
| Yamaoka et al. 1997 |
